# A Genome-Wide Integrative Study of DNA Methylation, Gene Expression, and Later Life Hand Grip Strength

**DOI:** 10.1093/geroni/igaa057.422

**Published:** 2020-12-16

**Authors:** Mette Soerensen, Jonas Mengel-From, Kaare Christensen, Lene Christiansen, Qihua Tan

**Affiliations:** 1 University of Southern Denmark, Odense C, Denmark; 2 University of Southern Denmark, Odense C, Syddanmark, Denmark; 3 Copenhagen University Hospital, Copenhagen Ø, Denmark

## Abstract

Hand grip strength (HS) measures muscular strength and associates with multiple health outcomes and mortality. Studies of epigenetic and transcriptomic markers could help elucidate the biology behind HS; markers for which monozygotic (MZ) twins are excellent study populations. We performed integrated enrichment analyses (IEA) of an epigenome-wide association analysis (EWAS) and a transcriptome-wide association analysis (TWAS) of HS in blood samples of 452 MZ twins (56-80 years of age). Unsupervised IEA were conducted by the KeyPathwayMiner algorithm, while supervised IEA were performed by the KEGG and Reactome databases. No individual CpG site or probe passed correction for multiple testing. Investigating the overlap in genes with p-values<0.01, 0.005 or 0.001 in the EWAS and TWAS, revealed 67, 21 and 2 unique genes, respectively. The latter 2 were TESK2 and VWA1. By the supervised approach, the 67-gene overlap identified three pathways related to “antigen processing and presentation”, driven by HLA-A, HLA-B, TAP2 and PSME2. With the unsupervised approach the 21-gene and 67-gene overlaps revealed networks containing 7 and 19 genes, respectively. Exception nodes (added by the algorithm for structure) were CREBBP and CSNK2A2 for the former, and APP and HSP90AB1 for the latter. The remaining IEA revealed no gene sets or networks. Several of these genes have previously been linked to HS relevant traits, e.g. arthritis (HLA-A, HLA-B and TAP2), smooth muscle and cardiovascular function (TESK2, HLA-B and APP) and sarcopenia (HSP90AB1). Hence, this study reports genes and pathways previously reported for physical functioning, yet also novel candidates for further verification.

